# Assessing the impact of a postnatal singing intervention on mental health, well-being measures and mother–infant interaction: results from the SHAPER-PND trial

**DOI:** 10.1136/bmjment-2026-302774

**Published:** 2026-10-12

**Authors:** Riddhi Laijawala, Rebecca H Bind, Sorcha Alford, Carolina Estevao, Katie Hazelgrove, Kristi Priestley, Lavinia Rebecchini, Madeline Kirkpatrick, Celeste Miller, Vaheshta Sethna, Anthony J Woods, Nikki Crane, Manonmani Manoharan, Alexandra Burton, Hannah Dye, Tim Osborn, Lorna Greenwood, Paola Dazzan, Carmine M Pariante, Daisy Fancourt

**Affiliations:** 1 Department of Psychological Medicine, Institute of Psychiatry Psychology & Neuroscience, King’s College London, London, UK; 2 Social Genetic and Developmental Psychiatry Centre, Institute of Psychiatry Psychology & Neuroscience, King’s College London, London, UK; 3 Culture Team, King’s College London, London, UK; 4 South London and Maudsley NHS Foundation Trust, London, UK; 5 Department of Behavioural Science and Health, University College London, London, UK; 6 Centre for Psychiatry and Mental Health, Wolfson Institute for Population Health, Queen Mary University of London, London, UK; 7 Breathe Arts Health Research, The Clarence Centre, London, UK; 8 Department of Psychosis Studies, Institute of Psychiatry Psychology & Neuroscience, King’s College London, London, UK

**Keywords:** Depressive Disorder, Mental Health, Music Therapy

## Abstract

**Background:**

Loneliness and a lack of social support are prominent risk factors in the development of postnatal depression (PND), affecting 24% of new mothers in the UK. Breathe Melodies for Mums (M4M), a 10-week group singing intervention, is effective at reducing depressive symptoms in PND; however, its impact on other clinical outcomes and mother–infant interaction remains unexplored.

**Objective:**

This study evaluated whether M4M, delivered within the Scaling-up Arts-Health Programmes: Implementation and Clinical Effectiveness Research-Postnatal Depression (SHAPER-PND) trial, improved mental health, well-being outcomes and mother–infant interaction, among mothers with PND.

**Methods:**

199 mothers were randomised 2:1 to a 10-week long singing (treatment group) or non-singing community activity (active control group). Mental health, well-being and social support were assessed at baseline, week 6 (midpoint), 10 (end of intervention), 20 and 36 (follow-up points) using the Hamilton Depression Rating Scale (HDRS) as well as self-report measures. Mother–infant interaction was assessed using the Infant Child Adult Relationship Experimental (CARE) Index at baseline, week 10 and week 36.

**Findings:**

Mothers in the singing group experienced a sharper decline in their depressive symptoms (HDRS: F(1,171)=9.27, p=0.003, ηp²=0.05) postintervention and reported improvement in social support (F(4,139)=5.93, p<0.001, ηp²=0.15). While all other secondary outcome measures improved gradually over time, there were no group effects or time × group interactions for stress, anxiety, well-being, loneliness or self-esteem. Both groups showed improvements in measures of mother–infant interaction, including dyadic synchrony F(2,150)=9.01, p<0.001, η^2^p=0.107.

**Conclusions:**

These findings underline the benefits of M4M, highlighting parallel improvements in social support. However, neither well-being nor mother–infant interaction appear to underlie these improvements.

**Clinical implications:**

Singing interventions, particularly M4M, remain as a promising adjunct treatment in the management of PND, requiring further confirmatory research.

**Trial registration number:**

NCT04834622.

**WHAT IS ALREADY KNOWN**

- Postnatal depression (PND) is a mental health condition estimated to affect 24% of new mothers in the UK. Evidence has found that a specifically tailored singing intervention for PND, Breathe Melodies for Mums (M4M), is clinically effective at reducing symptoms of depression up to 6 months beyond the end of sessions. However, potential benefits of this intervention beyond PND symptoms for new mothers have been less explored.

WHAT THIS STUDY ADDS

- This secondary outcome analysis investigates the impact of the same intervention on other dimensions such as clinician-rated depression, self-reported anxiety, loneliness, well-being and observer-rated mother–infant interaction. While both the singing and control group showed similar improvements on measures of anxiety, stress, loneliness and well-being, we did find that mothers who participated in the singing intervention experienced a greater reduction in severity of their depressive symptoms and felt a greater sense of social support by the end of the intervention period.

HOW THIS STUDY MIGHT AFFECT RESEARCH, PRACTICE OR POLICY

- This study demonstrates the benefits of community singing interventions for new mothers experiencing PND, with the potential of creative health interventions being used as treatment pathways during the perinatal period.

## Introduction

Postnatal depression (PND) is a mental health condition estimated to affect 24% of new mothers in the UK.^1^ Symptoms include feelings of sadness, heightened stress and anxiety, loss of interest and motivation, insomnia and feelings of guilt and worthlessness.^2^ In addition, PND is associated with decreased feelings of worth and self-efficacy.^3^ Two major contributors to the development of PND include lack of social support and overall feelings of loneliness and isolation,^4^ rates of which have heightened since the COVID-19 pandemic, driving increased rates of PND.^5^ PND has numerous downstream impacts on the offspring, which persist into childhood and adolescence such as impaired cognitive outcomes.^6^ However, one of the most immediate significant impacts of PND is on the mother–infant relationship,^7^ affecting both bonding and interaction.^8^ The mother–infant relationship is a critical tool in the first year of life, with disrupted interactions affecting cognitive and socioemotional outcomes.^9^

### Singing interventions for postnatal mental health and well-being

Although pharmacological treatment has had some positive results, this is hampered by low uptake, adherence among mothers and challenges while breastfeeding, while psychotherapy has produced mixed and short-lived results.^10–13^ However, given that studies examining predictors of PND have identified psychosocial factors such as daily hassles, parenting stress and both perceived and received social support,^14^ interventions that simultaneously reduce stress and enhance support networks could be of value. Recent studies, including our own, have highlighted the benefits of community-based arts-health interventions, such as singing, for symptoms of PND.^15–17^ A series of previous trials^16 18^ have found that a specifically tailored singing intervention for PND, Breathe Melodies for Mums (M4M) developed by Breathe Arts Health Research, is clinically effective at reducing symptoms of depression up to 6 months beyond the end of sessions^16^. However, potential benefits of this intervention beyond PND symptoms for new mothers have been less explored. In particular, there are two key gaps. First, in broader populations, community singing interventions have been demonstrated to impact outcomes such as anxiety and stress levels and overall sense of well-being, but whether similar benefits occur for mothers with PND is unclear.^19–21^ Second, we lack understanding of the impact of group singing on the mother–infant relationship.

### Singing for mother–infant bonding in PND

PND is best understood as a ‘relational disturbance’,^22^ and there is evidence to show that mothers with PND report less attachment security towards their infant and more fearful and preoccupied attachment types as compared with healthy controls.^23^ The COVID-19 pandemic has seen a stronger impact of maternal PND on the mother–infant relationship,^24^ with studies showing that higher levels of PND during the pandemic were associated with lower levels of mother infant attachment, up to 6 months postpartum.^25^ Additionally, associations between maternal depression and disorganised mother infant attachment have been observed in groups with higher socioeconomic disadvantage.^26^ However, evidence of the impact of singing on the mother–infant relationship is currently mixed. One study focusing on music and singing during pregnancy did not find significant effects on self-reported mother infant bonding, although the authors reported a significant increase in salivary oxytocin post intervention,^27^ which plays an important role in the mother infant relationship. In contrast, daily singing to babies postnatally has been associated with improvements in mother-infant bonding,^28^ while another study looking at mother–infant singing found significant increases in perceived mother–infant closeness in comparison to other mother and baby activities.^29^ In the context of preterm birth, music therapy has been portrayed as being no more effective at improving mother–infant bonding than standard care, although the differences in setting and population preclude direct inferences.^30^ Overall, evidence for the impact of arts in health interventions, especially singing, from a dyadic lens of mother–infant interaction is scarce.

### The SHAPER-PND study

This study is part of the Scaling-up Arts-Health Programmes: Implementation and Clinical Effectiveness Research-Postnatal Depression (SHAPER-PND) project; a hybrid type 2 randomised clinical trial, to assess the impact of a 10-week community singing intervention, M4M. The objectives and primary outcomes of the SHAPER-PND study have been previously published.^16^ The present paper has two objectives (1) to assess the effectiveness of the 10-week M4M intervention on overall maternal mental health, well-being and social support within the 10-week intervention period and up to 36-week follow-up and (2) to assess the effectiveness of the M4M intervention on mother–infant interaction within the same time frame.

## Methods

This paper is reporting on secondary outcomes from the SHAPER-PND trial, the protocol and primary outcomes of which have been previously published.^16 31^

### Study design

This was a multicentre trial across South London. Participants were randomised at baseline in a 2:1 allocation of singing intervention to active control groups. For equity of access, mothers in the control group were offered singing following the 10-week intervention period, outside of the study. All participants were followed during the study period at 6 and 10 weeks and then again at 20 and 36 weeks postbaseline. The trial registration number is **NCT04834622** (ClinicalTrials.gov, registered on 8 April 2021).

### Eligibility criteria

Inclusion criteria: mothers experiencing symptoms of PND, aged 18 or older; satisfactory understanding of English; with infants aged 0–9 months; with symptoms of depression who scored ≥10 on the Edinburgh Postnatal Depression Scale (EPDS)^32^ at screening (indicative of possible depression). The study was restricted to adults aged 18 and older as participation of teenage mothers would require additional ethics approvals and parental consent procedures. Exclusion criteria: unable to give informed consent or already partaking in another singing-based activity. This is with the caveat that exclusion criteria of other singing activities do not fully remove the potential confounding effect from other interventions or even standard care. To address this, information on attendance of psychological therapy and other mother and baby groups was collected and monitored during the course of the study, and have been reported in our primary outcomes paper.^16^

### Protocol changes since registration

We originally included meeting the criteria for a clinical diagnosis of major depressive disorder in the postnatal period (according to the Structured Clinical Interview for DSM-IV (SCID-IV) criteria) as an inclusion criterion; however, this greatly limited our recruitment abilities and we subsequently removed it as an official protocol deviation.

### Study setting and recruitment

Mothers were recruited by Breathe Arts Health Research through social media advertising, posters, clinics and other centres for mothers and their babies, signposting via health and social care professionals. Recruitment took place in London, with a particular focus in the South London boroughs of Lambeth, Southwark and Lewisham, as sessions were delivered in children’s centres and community venues in these areas.

A total of 255 mothers met screening eligibility and subsequently 199 mothers and their babies were fully eligible and enrolled to the trial across a total of seven cohorts of recruitment, from 20 September 2021 (recruitment of first cohort) to 31 July 2024 (end of study period for last cohort). A Consolidated Standards of Reporting Trials (CONSORT) participant flow diagram across the study period is presented in figure 1. In order to enrol in the trial, mothers filled an online form that included the EPDS. If their EPDS score was <10, they were notified that they were not eligible to participate and were signposted to other support services within the community (eg, talking therapies, mother–baby groups, baby activity groups). They were also notified that they could rescreen for eligibility at the next round of recruitment. If eligible (EPDS≥10), a member from Breathe would brief the participants about their eligibility, the study, next steps and put them in contact with the research team. Following this, participants were sent the Participant Information Sheet and Consent Form to review and to book in baseline assessments. Participants were then consented and enrolled into the trial, before completing their baseline assessment and being randomised into either the singing intervention or control group. Randomisation was performed by Sealed Envelope V.1.23,^33^ an online randomisation service, and followed a 2:1 intervention to control allocation process, following stratification by their EPDS eligibility score (10–13, 14–19, 20–30) and age of baby (0–3 months, 3–6 months, 6–9 months) in order to ensure balance across both groups. Given the nature of the intervention, both the mothers and research team (including assessors) were unblinded to group allocation. However, bias was handled through the use of standardised, validated measures, most of which were self-reported by participants. Two outcome assessors were present in participant assessments and reached consensus on observer-rated outcome measures such as the Hamilton Depression Rating Scale (HDRS). Finally, the intervention facilitators were not involved in data collection, and outcome assessors were not present in the singing sessions.

**Figure 1:**
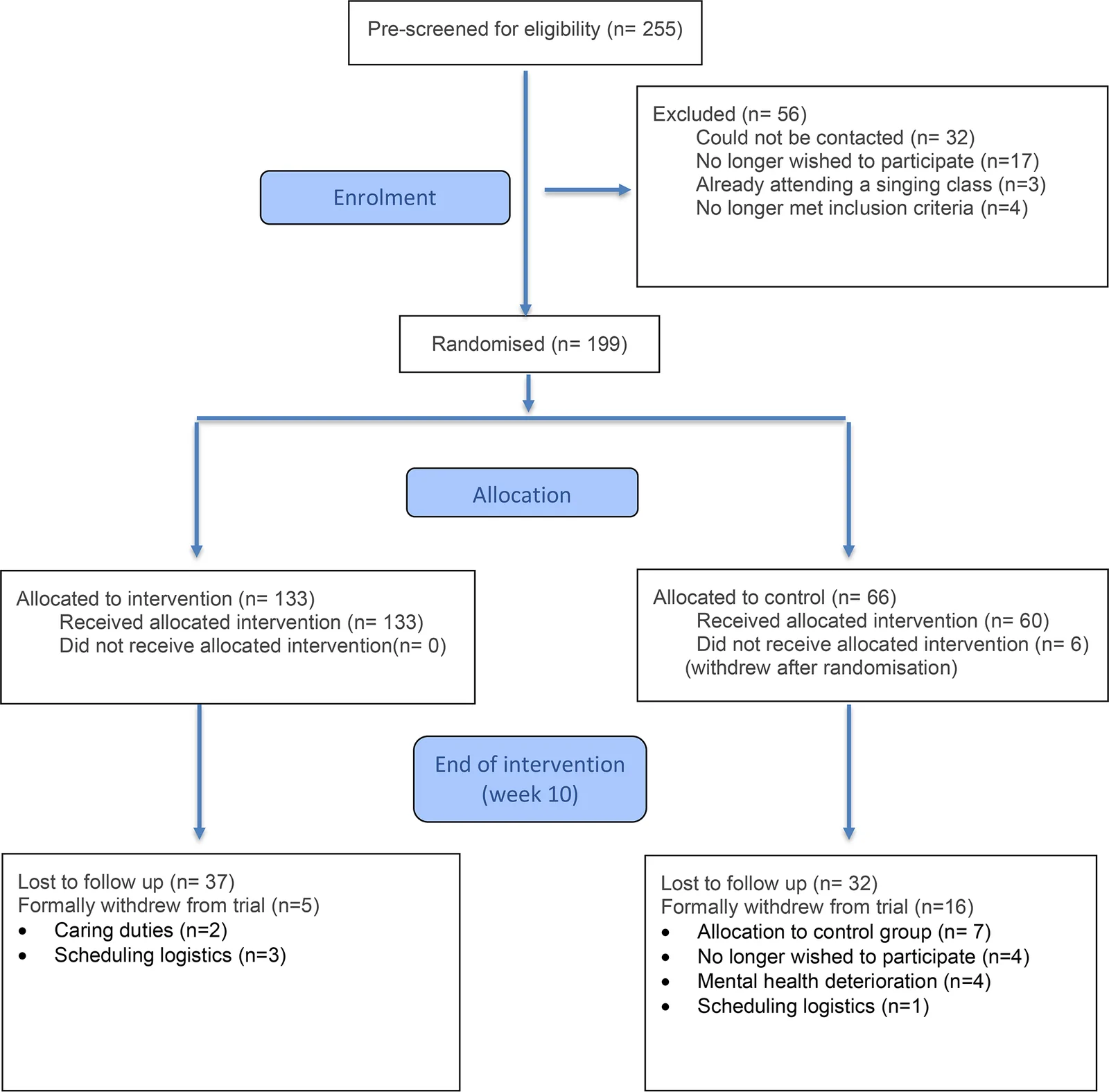
CONSORT 2025 flow diagram^64^

### Breathe M4M singing intervention group

Mothers and their babies who were allocated to the M4M intervention group attended 10 weekly in-person hour-long structured singing sessions, in groups of 8–12 dyads and continued with the same group and leader for the duration of the course. Sessions were led by a specialist Breathe-trained creative health music lead and a Breathe staff member trained in safeguarding and working with vulnerable people. Sessions took place on fixed days, at fixed timings and fixed locations, at Children’s Centres or other community venues. Attendance records were maintained by Breathe, and recordings of the songs were uploaded to private online platforms or onto CDs for the mothers to listen to at home. The dyads were not restricted from doing other activities for the rest of the week, with the caveat that none of these activities included a singing component. Full intervention details are available in our primary outcomes paper.^16^ Mothers and their babies sat in a circle on the floor surrounded by soft play cushions and mats. Classes began with welcome songs and icebreakers, introducing mothers and their babies to one another, and then involved teaching a range of songs from across the world in different languages. Songs were sung in rounds with multiple parts and harmonies and accompanied by maracas, drums and other simple instruments that mothers and their babies could play together. Mothers were reminded weekly by text or phone call of their session.

### Control group

Mothers in the ‘active’ control group were provided with details of other non-music mother-baby activities available in the community and received the same schedule of texts and phone calls as the intervention group to encourage them to join these activities. This provided an active control group so as to ascertain if there were benefits of offering bespoke M4M classes rather than encouraging mothers to engage in existing community provision.

### Secondary mental health, well-being and social support outcomes

Study data were collected and managed using REDCap electronic data capture tools hosted at King’s College London (version 15.0.36 on MacOS; Vanderbilt University, Nashville, Tennessee, USA; https://project-redcap.org/)^34^

#### Demographics

At baseline, we collected demographics as well as measures of risk factors for PND and difficulties in the mother-infant relationship: the Threatening Life Experiences Questionnaire^35^; the Childhood Experience of Care and Abuse Questionnaire (CECA.Q)^36^; the Composite Abuse Scale (CAS)—Pregnancy Version^37^; and the Intrusive Life Events Scale.^38^

#### Secondary mental health outcomes

We collected via semi-structured interview the HDRS^39^ at baseline and week 10 (end of intervention) to assess for change in severity of depressive symptoms. Additionally, in order to understand the trajectory of further symptoms of depression, anxiety and stress, we collected via online self-report questionnaire at baseline and weeks 6, 10, 20 and 36: the Beck Depression Inventory (BDI),^40^ the State-Trait Anxiety Inventory—State (STAI-S),^41^ the Office of National Statistics—Anxiety subscale^42^ (ONS-A) and the Perceived Stress Scale (PSS).^43^

#### Secondary well-being and social support outcomes

To assess whether the M4M intervention was associated with improved well-being and perceived social support, we collected via online self-report questionnaire at baseline and weeks 6, 10, 20 and 36: the University of California, Los Angeles (UCLA) Loneliness Scale,^44^ the Short General Self-Efficacy Scale (GSE-6),^45^ the Office of National Statistics–Worthwhile, Satisfaction and Happiness subscales (ONS-W, ONS-S, ONS-H),^42^ and the Multidimensional Scale of Perceived Social Support (MSPSS).^46^

#### Measurement of mother–infant interaction

To assess mother–infant interaction, we used the Child Adult Relationship Experimental (CARE) Index^47^; a validated measure that enables researchers and clinicians to rate interactions from a dyadic perspective recommended by the Royal College of Psychiatrists as part of a framework for routine outcome measures in perinatal psychiatry.^48^ The CARE Index is a valid and reliable method of evaluating adult–infant interaction patterns, used in screening, research and intervention contexts.^7 47^ We filmed 3 minute interactions over Zoom at Baseline, week 10 and week 36 and instructed the mothers to interact with their baby as they normally would. The interaction was assessed based on seven aspects of behaviour: facial expression, vocal expression, position and body contact, affection and arousal, turn taking contingencies, control, and choice of activity. Based on scores given across these aspects, the interaction was rated to assess maternal behaviour, infant behaviour and overall dyadic synchrony. There are different dimensions within this relating to maternal and infant behaviour patterns, but the present study reports only on dyadic synchrony, maternal unresponsiveness, infant passivity and infant difficultness, since they have previously been reported in the literature in the context of antenatal depression.^7^ Therefore, we build on this evidence and investigate the same constructs in the context of PND. The interaction is scored in terms of dyadic synchrony, a construct measuring how in tune the dyad is^7^ and how ‘optimal’ the overall interaction is. This is rated from 0 to 14, with higher dyadic synchrony reflecting more positive interactions. Maternal unresponsiveness is a pattern indicating being unconnected or withdrawn from the interaction. Infant compulsivity is a pattern presenting as frozen watchfulness, fear and compliance. Finally, infant passivity is a pattern reflecting as unconnectedness, low arousal and withdrawal from the interaction. Both infant and maternal behaviour patterns are scored from 0 to 14, and higher scores indicate greater prevalence of the pattern. Dyadic synchrony scores are understood within the context of the need for intervention. Scores from 0 to 4 are placed into the ‘risk’ category (a need for parental psychotherapy with potential separation of the dyad), 5–6 in the ‘inept’ category (voluntary parent–infant intervention), 7–10 being ‘adequate’ (no intervention necessary, optional parental education), and finally, 11–14 being ‘sensitive’ (no intervention necessary).^7^

The trained raters (RL and SA) have achieved level III+reliability with the CARE Index course. Additionally, they have achieved internal reliability, with a mean interclass correlation of 0.81 across all scales (see online supplemental figure 1 for participant flowchart for video coding, including the number of those consented, assessed at each time point and included in the final analysis).

### Statistical analysis

Analyses were conducted with SPSS Statistics V.29–31 for MacOS (IBM, UK). Before analysis, data were checked for normality. Demographics were analysed using frequencies and descriptives and are reported as mean (M) and SD, and as frequency (n) and percentage (%) for categorical variables. Independent samples t-tests were used to compare groups for continuous data, while the Pearson’s χ^2^ was used to compare groups for categorical outcomes. Trajectory of mental health, well-being and social support outcomes were analysed using Repeated Measures Analysis of Variance (ANOVA) with 95% CIs and least significant difference (LSD) corrections for post-hoc analysis to examine differences at each time point, which was exploratory. While we acknowledge that LSD corrections are interpreted with limited caution, posthoc findings were consistent with ANOVA findings and our primary outcome results, improving confidence in the robustness of the results. Due to group differentials in missing data, whereby the control group had a significantly higher rate of missing data likely due to group allocation, data were analysed with an intention to treat approach using last observation carried forward for missing data. This approach was also used in our primary outcome paper.^16^ We acknowledge that different measures have different numbers of participants included, this is represented by the different df for each statistical test.

The CARE-Index was assessed at baseline, week 10 and week 36. Given that repeated measures ANOVA requires complete data across timepoints, participants with missing data at any timepoint were excluded listwise. These data were tested for normality and all CARE Index data were log transformed. On log transformation, the data still violated normality, but given the robustness of repeated measures ANOVA to moderate non-normal samples, and with our sample size ≥30 in both groups,^49^ we conducted this analysis with 95% CIs and effect sizes reported as partial η_p_^2^. Posthoc pairwise comparisons were conducted in an exploratory manner to identify at which timepoints the randomisation groups differed, using the LSD procedure. To account for the fact that fewer participants had videos at all three timepoints than at baseline and week 10 alone, we did two separate ANOVAs, the first with complete data at baseline, week 10 and week 36 (77 in total, 63 in the singing group and 14 in the control group), and the second for baseline and week 10 only (108 women in total, 78 in the singing group and 30 in the control group).

For all analyses, assumptions of ANOVA were checked in SPSS. Q-Q plots of residuals indicated normality, and Mauchly’s test of sphericity confirmed that this indeed was met (p>0.05). Therefore, no meaningful violations of assumptions were observed. Further details on methodology can be found our primary outcomes paper.^16^ Applying 0.05/(14 (total number of outcome measures included)), a Bonferroni alpha of 0.00357 was calculated, indicating that results with a p value below this were robust to multiple testing. Finally, linear mixed effect models were fitted as sensitivity analyses to test the robustness of our primary ANOVA analyses, to assess the consistency of effects using two statistically different approaches. The model included treatment group, time, and time × group interactions as fixed effects. Restricted maximum likelihood was used, and the time × group interaction was used to assess whether changes in the included outcomes over time differed across the singing and control groups.

## Results

199 mothers were included in the main analysis. 108 mothers had videos at 2 timepoints (baseline and week 10) and 77 mothers (mean age 35 years) who had videos at all 3 timepoints (baseline, week 10 and week 36). The sociodemographic characteristics of participants in both groups are presented in online supplemental table 1.

### Postnatal depressive symptoms

We explored the HDRS mean scores between baseline and week 10 (end of intervention) and the trajectory of mean scores on the BDI between baseline and week 36 (end of study).

For the HDRS, while on average mothers experienced an improvement in symptoms over time in both the singing and control groups (time: *F(1,171)= 75.27, p<0.001, ηp²=0.31),* mothers in the singing group experienced a sharper decline in their symptoms, suggesting the singing intervention was more effective in reducing the severity of mothers’ depression by the end of intervention *(time × group interaction: F(1,171)=9.27, p=0.003, ηp²=0.05)* (figure 2). In our sensitivity analysis, a linear mixed-effects model demonstrated significant time x group interactions, (F(1,171)= 9.272, p=0.003), confirming our primary ANOVA analysis.

**Figure 2:**
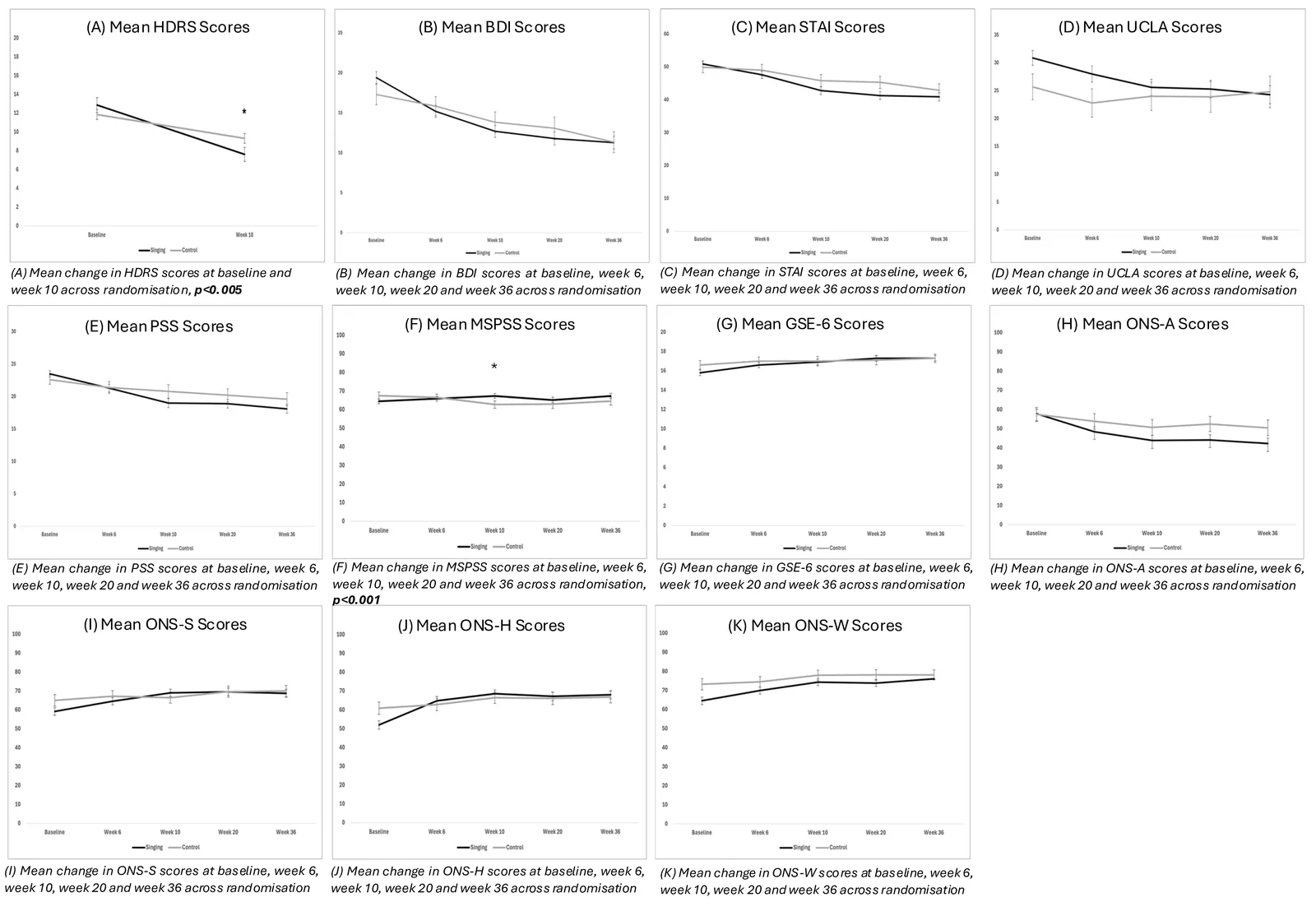
Mean change in HDRS (A), BDI (B), STAI (C), UCLA (D), PSS (E), MSPSS (F), GSE-6 (G) and ONS (H,I,J,K) scores across randomisation. BDI, Beck Depression Inventory; GSE-6, Short General Self-Efficacy Scale; HDRS, Hamilton Depression Rating Scale; MSPSS, Multidimensional Scale of Perceived Social Support; ONS, Office of National Statistics; ONS-H, ONS—Happiness; ONS-S, ONS—Satisfaction; ONS-W, ONS—Worthwhile; PSS, Perceived Stress Scale; STAI, State-Trait Anxiety Inventory; UCLA, UCLA Loneliness Scale.

Using the BDI across the whole study period (baseline to week 36), we found a similar improvement in symptoms across both groups (time: *F(4,135)=23.72, p<0.001,* ηp²*=*0.41), but unlike the HDRS, we did not find a significant time × group interaction at any time point, indicating that mothers in both groups experienced similar reductions in their perceived symptoms of depression. In our sensitivity analysis, a linear mixed-effects model demonstrated no significant time × group interactions, (F(4,579.15)=2.09, p=0.08), confirming our primary ANOVA analysis.

### Postnatal anxiety and stress symptoms

Using all three measures of anxiety and stress across the whole study period (baseline to week 36), we found an overall effect of time (STAI-S: *F(4,142)=12.03, p<0.001, ηp²=0.25; ONS-A: F(4,138)=5.42, p<0.001, ηp²=0.14; PSS: F(4,148)=16.19, p<0.001, ηp²=*0.30) but no significant main effect of group or interaction at any time point, suggesting that mothers in both groups experienced similar reductions in their symptoms of anxiety and stress (see figure 2). In our sensitivity analysis, a linear mixed-effects model demonstrated no significant time × group interactions, (STAI-S: F(4,590.23)=1.14, p<=0.33; ONS-A: F(4,575.163)=0.93, p=0.44; PSS: F(4,612.58)=1.99, p=0.093), confirming our primary ANOVA analysis.

### Social support

We did not find an overall effect of time on mothers’ level of perceived social support between baseline and week 36. However, we did find a significant group × time interaction, whereby mothers in the singing group reported feeling a greater sense of social support across time than mothers in the control group (*F(4,139)=5.93, p<0.001, ηp²=0.15).* Exploratory post-hoc analyses revealed these differences to have occurred at week 10, overall suggesting that by the end of the intervention period, mothers in the singing group experienced a greater sense of social support than mothers who participated in the control activities (see figure 2). In our sensitivity analysis, a linear mixed-effects model demonstrated significant time × group interactions (F(4,578.31)=3.83, p=0.004), confirming our primary ANOVA analysis.

### Well-being

For all well-being measures used, we found a time effect, with improvements in both the singing and control groups (ONS: *F(4,139)=6.36, p<0.001, ηp²=0.16;* worthwhile: *F(4,140)=6.89, p<0.001, ηp²=0.16;* happiness: *F(4,143)=6.56, p<0.001, ηp²=0.16;* UCLA: *F(4,120)=3.29, p=0.014, ηp²=0.10;* GSE-6: *F(4,144)=7.44, p<0.001, ηp²=0.17).* However, there were no main effects of group or time × group interactions (see figure 2). In our sensitivity analysis, a linear mixed-effects model demonstrated no significant time × group interactions (ONS satisfaction: F(4,602.96)=2.27, p<=0.06; worthwhile: F(4,601.97)=1.45, p=0.21; UCLA: F(4,579.25)=1.77, p=0.13, GSE-6: F(4,613.39)=0.908; p=0.459), confirming our primary ANOVA analysis. In this linear mixed effects model analysis, only the happiness subscale of the ONS had a significant time × group interaction (F(4,580.45), p=0.03).

### Mother–infant interaction

We found an overall effect of time (across three timepoints) indicating improvements in both groups on dyadic synchrony F(2,150)=9.01, p<0.001, η^2^p=0.107), maternal unresponsiveness F(2,150)=3.07, p<0.05, η^2^p=0.039) and infant passiveness F(2,150)= 4.14, p<0.05 η^2^p=0.052), but no group or time × group interactions. We did not find any time effects for infant compulsivity F(2,150)= 0.966, p=0.38, η^2^p=0.013) (see figure 3). In our sensitivity analysis, a linear mixed-effects model demonstrated no significant time × group interactions for dyadic synchrony (F(2,196.98)=0.89, p=0.411), maternal unresponsiveness (F(2,190.45)=0.56, p=0.57), infant passiveness (F(2,198.25)=0.23, p=0.098) and infant compulsivity (F(2,203.81)=0.102, p=0.903), confirming our primary ANOVA analysis.

**Figure 3:**
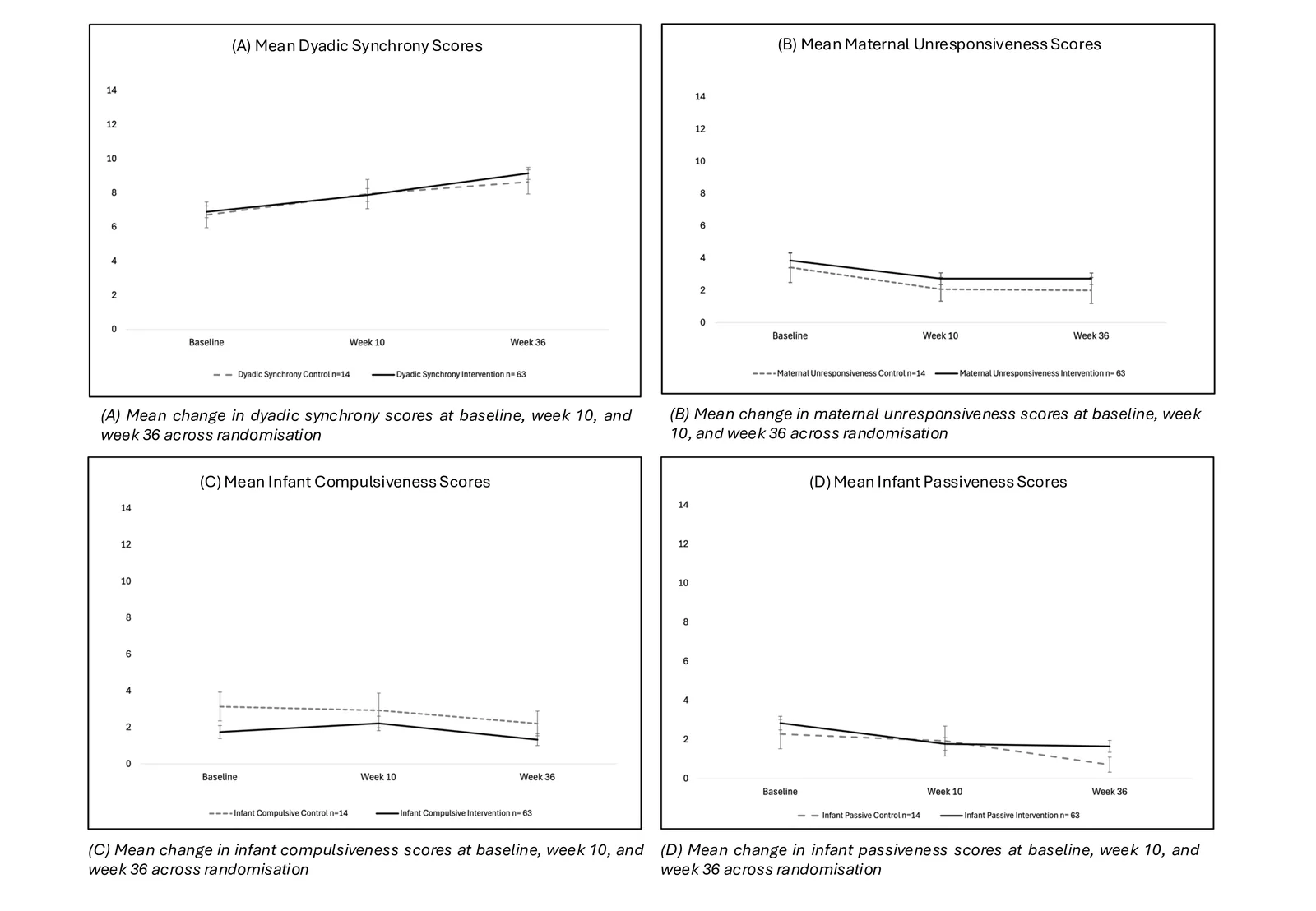
Mean change in CARE index variables across randomisation. CARE, Child Adult Relationship Experimental Index.

Additional results on analysis of Baseline and week 10 are found in online supplemental material 1).

See online supplemental table 2 for means and SD of all included variables across randomisation.

## Discussion

This study expands on previous clinical trials^15–18 50 51^ on a bespoke singing intervention for mothers with PND to consider a wide range of secondary outcomes including further aspects of depression, anxiety, stress, well-being, social support and mother–infant relationship. While both the M4M and control group showed similar improvements on measures of anxiety, stress, loneliness and well-being, we did find that, compared with the control group, mothers who participated in the singing intervention experienced a greater reduction in severity of their depressive symptoms (measured by the clinician-rated HDRS) and felt a greater sense of social support (using the MSPSS) by the end of the intervention period. Further qualitative work from the study^52^ has identified core ingredients such as (a) artistic content involving diverse ranges of songs in a group, (b) social composition involving small groups of mums going through similar experiences and (c) activity facilitation involving skilled and inclusive musical leads and experienced Breathe staff, all of which highlight how the M4M intervention can address low uptake and adherence in perinatal care

In terms of depression, while mothers in both the M4M and control group experienced an overall decrease in the severity of their symptoms on the HDRS by week 10, participating in the M4M intervention led to a significantly sharper decline in symptoms. In fact, by week 10, on average, mothers in the M4M group were rated as no longer depressed according to scoring criteria, while mothers in the control group were (on average) rated as still experiencing mild depression, highlighting the quick-acting and profound effects of M4M on PND. These results are in line with a recent systematic review, which found that singing interventions are more effective than control activities at reducing symptoms of PND^53^ and fit with our study’s primary findings, which showed that mothers who participated in M4M had lower depressive symptoms on the EPDS at week 10 following the intervention than mothers in the control group.^16^ Interestingly, we did not find the same effect of M4M on the BDI, whereby mothers in both groups reported comparable decreases in their depressive symptoms at all time points. It is important to note that the BDI is a self-rated questionnaire while the HDRS is a clinician-rated interview and they assess different aspects of depression: while the BDI is more centred on subjective feelings, the HDRS focuses on objective symptoms. In fact, previous studies have also found discrepancies between the two scales in how they evaluate change in symptoms,^54^ and in the field of antenatal depression, Laijawala *et al*^55^ found differences in treatment efficacy based on self-reported, versus clinician-rated measures. Thus, it is possible that M4M may be more effective than control activities at specifically tackling somatic symptoms associated with depression, particularly because the act of singing is associated with physical relaxation and stress reduction.^29^

With regard to perceived social support, mothers in the M4M group reported a significantly greater sense of social support by the end of the intervention period. These findings are consistent with qualitative studies that have reported improvements in social connections and support from mothers engaged in the M4M intervention.^15 56^ Given that a lack of social support is a significant risk factor for the development of PND, it is to be expected that mothers’ increase in social support was coupled with a decrease in their depressive symptoms. There are likely various mechanisms involved in singing that lead to improvements in social support: for example, Breathe Arts Health Research specifically designed the M4M intervention with social connection in mind, such that mothers were sat in a circle together and eye contact through the singing of rounds, call and response patterns of vocal activity, and group coordination was encouraged throughout each song. All of these factors foster continuous interactions and social bonding within a group and thus may not be present in other mother–baby activities that instead place focus on promoting activity within individual mother–baby pairs.^57^ Additionally, previous studies have found that the act of singing promotes the release of oxytocin and endorphins, both of which are heavily involved in social connection and bonding.^58^ Finally, it is thought that participation in activities which promote synchrony between individuals, like group singing, leads to greater feelings of belonging, which in turn is likely to increase subsequent attendance at the activity.^59^ This is supported by the fact that mothers in the M4M group attended more sessions than mothers in the control activities and were less likely to withdraw from the study.^16^

In terms of anxiety, stress and overall well-being, mothers in both the M4M and control groups improved across the study period, with no group differential in rate of improvement. However, it is important to note that this study involved an active control: mothers in the control group were in close contact with the study team throughout the 10-week intervention period, received support and safeguarding when necessary and were encouraged to attend local mother–baby activities when they might otherwise have not engaged in a group activity. Consequently, these shared ingredients across intervention and control groups could help to explain improvements in anxiety, stress and well-being during the initial 10 weeks. It does suggest that methods like signposting, scaffolding, support and regular contact could be equally effective for improving those outcomes, although it is important to note that the primary outcome of this trial—PND symptoms—still appears to be more effectively and sustainably improved through M4M, with greater cost-effectiveness, acceptance, feasibility and appropriateness too.^16^

With regard to mother–infant interaction, we found that dyadic synchrony improved over time in both groups, and we did not find a significant time×group interaction, indicating that dyads experienced similar improvements in dyadic synchrony regardless of their randomisation. However, it is important to note that in this overall sample of postnatally depressed women, dyadic synchrony scores in both groups were in the ‘adequate’ range; that is, they indicated a relatively good quality of interaction. In comparison, when looking at other similar samples of mothers with depression starting in pregnancy, dyadic synchrony scores were in the Risk or Inept categories.^7^ This highlights a key finding that perhaps it is depression experienced in pregnancy which has a more profound impact on mother–infant interaction, as compared with PND alone. Similar findings have been reported, wherein women experiencing high levels of depression in pregnancy, but not postnatally are less responsive towards their infants,^60^ while infants of mothers who are antenatally depressed demonstrate a disorganised attachment style.^61^ Interestingly, findings from Rebecchini *et al* from the same cohort explored another construct of the mother–infant relationship, that is, mentalisation and communication.^50^ Mothers randomised to the singing group showed significantly higher proportions of mentalising comments and infant-focused speech, and fewer negative comments, when compared with the control group. Of note, the proportion of mentalising comments at the beginning of the study in our sample (around 0.02–0.03) are similar to those described in the aforementioned study in antenatal depression and much lower than the average proportion in women with no depression (approximately 0.07).^62^ Thus, it is possible that the reason that we are seeing an improvement in mentalisation (but not the specific domains of mother–infant interaction) is that mentalisation was affected by PND, and therefore more likely to improve in parallel with the improvement of depression, which was larger in the singing group. We, therefore, postulate that our singing intervention also differentially influences distinct, yet interconnected constructs of the mother–infant relationship. In contrast, the mother–infant interaction, because it was not impacted by PND, was less likely to improve in parallel with the improvement in depression. Of course, the preserved mother–infant interaction in our sample might be explained by the fact that the inclusion criteria for our project are EPDS ≥10, indicative of ‘possible’ or ‘mild’ depression.^16^ Existing literature focusing on severe PND in a group of mothers in an inpatient Mother and Baby Unit has found that infants of mothers with unipolar depression presented as having insecure attachment styles, while the mothers have been observed to be less sensitive and more negative in their play interactions.^63^ However, we acknowledge that the evidence is mixed, seeing as previous work has found that women with moderate–severe levels of depression experience faster improvements.^18^

We acknowledge that the active control group was exposed to several non-music activities already available in the community. As the aim of the M4M study was to isolate the impact of singing on PND, the control group was designed to participate in a range of existing non-music community activities for comparison. Although the diversity of these activities may have introduced some heterogeneity within the control group, the main findings discussed in our primary outcomes paper^16^ indicate that the singing intervention was more suitable and acceptable in addressing postnatal depressive symptoms than the control activities.

We found similar improvements in the singing and active control groups on outcome measures such as loneliness, anxiety, stress and mother–infant interaction. It is likely that the heterogeneity of control group conditions along with the standard of usual care also led to overall improvements. However, as shown in our primary outcomes, the benefit of singing was sustained up to 6-month postintervention on symptoms of self-reported depression, which was not the case in control group activities.^16^ This, along with the additional cost–benefit of M4M^16^ makes the intervention overall suitable in the treatment of PND.

The results of our analysis need to be interpreted in the context of certain limitations. The findings presented here might be explained by the relatively mild nature of the PND for inclusion in the study and by the sample size, as we fell short of the initial recruitment target of 400 women due to the COVID-19 pandemic as the usual in-person recruitment pathways that reach the most mothers were restricted. This, along with the attrition of 69 participants, meant that our study was underpowered. Nevertheless, our sample provided an expansion from the previous RCT (199 participants across two arms here vs 134 across three arms previously).^18^ As a recruitment-related limitation, we acknowledge that recruitment via advertisement is likely to introduce bias. However, for this reason, we also used other methods such as clinics and signposting via healthcare professionals to allow for wider representation. A larger study involving more severe cases of PND might have identified additional differential effects of the singing intervention on broader measures, alongside the important differences in clinician-rated depression and social support that we do find. Furthermore, we acknowledge that the HDRS was not repeated at follow any up points (week 20 or week 36). As such, we cannot determine whether the clinician-rated advantage persisted at 20 or 36 weeks, nor fully interpret the discrepancy between HDRS and BDI trajectories. Additionally, only a subsample of the mothers provided videos for mother–infant interaction analyses, meaning sample bias may have affected results. However, it is notable that there was no meaningful difference in the demographics between the two samples. There was the opportunity to rescreen in the future after a period of at least 1 month, potentially introducing the possibility of screening as more severe so as to meet inclusion criteria. However, as per study records no women actually rescreened if they were not eligible in the first instance. Therefore, there was no possibility of confounding or bias due to this. Finally, while we monitored to ensure that mothers in the control group were not involved in weekly singing classes during the first 10 weeks of the programme, after this, mothers in both groups were encouraged to participate in any community activities, including musical ones. In this study, we did not monitor behavioural patterns of musical engagement in this follow-up period. But future studies are encouraged to explore this further, and to consider how 10 weeks of singing for the experimental group may have influenced subsequent patterns of musical engagement. In considering other opportunities for future research, infant behavioural outcomes were not assessed in our study, and, therefore, implications for socioemotional offspring outcomes remain to be determined.

Nevertheless, our results demonstrate valuable insights into understanding the impact of a singing intervention on depression measured with a clinician-rated instrument. In particular, we confirm, using a clinical diagnostic scale, that the singing intervention is more effective than the control by the end of the 10-week treatment in reducing PND symptoms and shows increased perceived social support. These findings build on previous analyses from the SHAPER study on efficacy and implementation.^15–17 31 50 51^ Furthermore, the findings feed into an emerging overarching theory of change for how M4M impacts symptoms of PND in mothers. This work has established M4M’s core active ingredients (including the specific parameters of the activity, the social exposure provided and the therapeutic environment) and is gradually building an understanding of how M4M works by activating psychological mechanisms (eg, reduced stress and anxiety, enhanced self-confidence, positive emotions, purpose and identity), biological mechanisms (eg, reduced cortisol and inflammation, improved mother–infant physiological synchrony),^51^ social mechanisms (eg, reduced isolation and loneliness, stronger peer support and mother–infant communication),^50^ and behavioural mechanisms (eg, increased singing at home, positive routines, parenting self-efficacy and motivation), while also supporting infant well-being through reductions in crying, colic and sleep disturbances. The findings presented here elucidate that while some of these mechanisms can also be activated by other community mother–infant classes, the sense of social support provided by M4M as an intervention appears particularly important. Overall, this work is providing an extended framework that offers insight into community-based arts intervention to support perinatal mental health.

## Supplementary material

10.1136/bmjment-2026-302774 online supplemental file 1

10.1136/bmjment-2026-302774 online supplemental figure 1

10.1136/bmjment-2026-302774 online supplemental table 1

10.1136/bmjment-2026-302774 online supplemental table 2

## Data Availability

Data are available upon reasonable request.
