# Supplementary material for: Assessing the impact of a postnatal singing intervention on mental health, well-being measures and mother–infant interaction: results from the SHAPER-PND trial
Source: BMJ Ment Health. 2026 Oct 12;29(1):e302774. doi: 10.1136/bmjment-2026-302774 (PMC13662075; doi:10.1136/bmjment-2026-302774)
Supplement: Supplementary file 1 [file bmjment-29-1-s002.docx]

Figure 1-SHAPER-PND study participant flow chart for CARE-Index video coding
