# Supplementary material for: Assessing the impact of a postnatal singing intervention on mental health, well-being measures and mother–infant interaction: results from the SHAPER-PND trial
Source: BMJ Ment Health. 2026 Oct 12;29(1):e302774. doi: 10.1136/bmjment-2026-302774 (PMC13662075; doi:10.1136/bmjment-2026-302774)
Supplement: Supplementary file 1 [file bmjment-29-1-s001.docx]

**Supplementary material 1**

## Mother infant interaction analysis- Baseline to Week 10

We found an overall effect of time in both groups, from baseline to week 10 (end of the intervention) for maternal unresponsiveness F(1,106) = 5.34, p<0.05, η^2^p=0.048), but no group or time x group interactions. We did not find any time effects for dyadic synchrony F(1,106)=0.82, p=0.36, η^2^p=0.008, infant compulsivity F(1,106)= 1.79, p=0.18, η^2^p=0.017), and infant passiveness F(1,106)= 1.87, p=0.18, η^2^p=0.017).
