# Supplementary material for: Assessing the impact of a postnatal singing intervention on mental health, well-being measures and mother–infant interaction: results from the SHAPER-PND trial
Source: BMJ Ment Health. 2026 Oct 12;29(1):e302774. doi: 10.1136/bmjment-2026-302774 (PMC13662075; doi:10.1136/bmjment-2026-302774)
Supplement: Supplementary file 3 [file bmjment-29-1-s003.docx]

| Variable | Secondary outcomes analysis | | | Mother infant interaction analyses | | |
| --- | --- | --- | --- | --- | --- | --- |
|  | Singing Group | Control Group | Statistic | Singing Group | Control Group | Statistic |
| Maternal age in years M (SD) | 35.6 (4.4) | 35.0 (4.9) | P = 0.43 | 35.5 (4.5) | 34.6 (4.7) | P = 0.37 |
| Infant age in months M (SD) | 3.6 (2.7) | 4.0 (2.5) | P = 0.36 | 4.1 (2.4) | 4.2 (2.5) | P=0.85 |
| Infant sex, n (%)- |  |  |  |  |  |  |
| Female | 61 (52.6) | 28 (50.0) | P = 0.87 | 35 (45.5) | 15 (50) | P=0.67 |
| Male | 55 (47.4) | 28 (50.0) |  | 42 (54.5) | 15 (50) |  |
| Maternal relationship status n (%) |  |  |  |  |  |  |
| Married or cohabiting | 103 (88.0) | 42 (73.7) | P = 0.029 | 67 (76.1) | 21 (23.9) | P= 0.057 |
| Single with or without a partner | 14 (12.0) | 15 (26.3) |  | 11 (55) | 9 (45) |  |
| Maternal ethnicity, n (%) |  |  |  |  |  |  |
| Any White background | 83 (70.9) | 30 (52.6) | P = 0.027 | 55 (72.4) | 23 (71.9) | P= 0.95 |
| Ethnic minority groups | 34 (29.1) | 27 (47.4) |  | 21 (27.6) | 9 (28.1) |  |
| Maternal qualifications, n (%) |  |  |  |  |  |  |
| Higher education or above | 103 (88.0) | 48 (84.2) | P = 0.48 | 69 (88.5) | 27 (90) | P= 0.8 |
| GCSE/A- level or below | 14 (12.0) | 9 (15.8) |  | 9 (11.5) | 3 (10) |  |
| Maternal employment status n (%) |  |  |  |  |  |  |
| Employed/student/maternity leave | 108 (92.3) | 52 (91.2) | P = 0.78 | 69 (88.5) | 27 (90) | P=0.82 |
| Unemployed/full-time mother | 9 (7.7) | 5 (8.8) |  | 9 (11.5) | 3 (10) |  |
| Household income, n (%) – (6 missing) |  |  |  |  |  |  |
| <30,000 | 12 (10.6) | 12 (23.1) | P = 0.06 | 64 (85.3) | 20 (74.1) | P=0.18 |
| ≥30,000 | 101 (89.4) | 40 (76.9) |  | 11 (14.7) | 7 (25.9) |  |
