# Supplementary material for: Assessing the impact of a postnatal singing intervention on mental health, well-being measures and mother–infant interaction: results from the SHAPER-PND trial
Source: BMJ Ment Health. 2026 Oct 12;29(1):e302774. doi: 10.1136/bmjment-2026-302774 (PMC13662075; doi:10.1136/bmjment-2026-302774)
Supplement: Supplementary file 4 [file bmjment-29-1-s004.docx]

|  |  | Singing group | |  |  |  | Control group | |  |  |
| --- | --- | --- | --- | --- | --- | --- | --- | --- | --- | --- |
|  | **Baseline**  **(M±SD)** | **Week**  **6**  **(M±SD)** | **Week**  **10**  **(M±SD)** | **Week**  **20**  **(M±SD)** | **Week**  **36**  **(M±SD)** | **Baseline**  **(M±SD)** | **Week**  **6**  **(M±SD)** | **Week**  **10**  **(M±SD)** | **Week**  **20**  **(M±SD)** | **Week**  **36**  **(M±SD)** |
| HDRS | 12.9±5.5 |  | 7.6±5.5 |  |  | 11.9±5.9 |  | 9.3±6.0 |  |  |
|  |  |  |  |  |  |  |  |  |  |  |
| BDI | 19.4±7.4 | 15.2±7.3 | 12.7±7.64 | 11.8±8.3 | 11.3±8.1 | 17.3±7.6 | 15.8±8.2 | 13.8±7.6 | 13.1±8.3 | 11.3±8.2 |
|  |  |  |  |  |  |  |  |  |  |  |
| STAI | 50.9±10.5 | 47.5±11.1 | 42.8±12 | 41.3±12.3 | 40.8±13 | 49.8±9.65 | 49±12.67 | 45.7±12.6 | 45.3±12.3 | 42.9±12.9 |
|  |  |  |  |  |  |  |  |  |  |  |
| PSS | 23.5±4.5 | 21.3±5.6 | 19±6.6 | 18.9±6.82 | 18.1±6.6 | 22.6±5.9 | 21.3±7.5 | 20.8±8.05 | 20.2±6.9 | 19.6±7.5 |
|  |  |  |  |  |  |  |  |  |  |  |
| UCLA | 30.8±13 | 28±14 | 25.6±14.2 | 25.3±15.3 | 24.2±16 | 25.7±12.6 | 22.7±16 | 23.9±13.8 | 23.9±14.9 | 24.7±15.1 |
|  |  |  |  |  |  |  |  |  |  |  |
| GSE-6 | 15.8±3 | 16.5±2.9 | 16.9±2.9 | 17.2±3.2 | 17.3±2.9 | 16.5±2.8 | 17±2.9 | 16.9±3.3 | 17.1±3.2 | 17.2±2.8 |
|  |  |  |  |  |  |  |  |  |  |  |
| MSPSS | 64.5±12.5 | 65.9±11.6 | 67.4±11.5 | 65.2±14.4 | 67.3±13.8 | 67.6±12.6 | 66.6±14.7 | 62.8±16.5 | 63±16.4 | 64.5±15.1 |
|  |  |  |  |  |  |  |  |  |  |  |
| ONS-A | 57.7±23.1 | 48.4±26.1 | 43.9±27.2 | 44.2±26.3 | 42.3±26.2 | 57.5±26.4 | 53.8±27.6 | 50.7±28.2 | 52.5±26.7 | 50.5±30.7 |
|  |  |  |  |  |  |  |  |  |  |  |
| ONS-S | 59.2±19.8 | 64.5±19.2 | 68.9±18.1 | 69.5±19.6 | 68.7±20 | 65.1±21.3 | 67.3±19.9 | 66.5±22.4 | 69.6±19.3 | 69.6±21 |
|  |  |  |  |  |  |  |  |  |  |  |
| ONS-W | 64.6±20.3 | 70±17.4 | 74.4±16.7 | 73.8±18 | 76.1±17.6 | 73.3±19.1 | 74.5±20.8 | 77.9±20 | 78.2±17.9 | 78.2±18.3 |
|  |  |  |  |  |  |  |  |  |  |  |
| ONS-H | 52±21.7 | 64.9±19.9 | 67.2±22 | 68.1±20.5 | 60.8±23.9 | 62.7±25.9 | 62.7±25.9 | 66.5±23.4 | 66.1±23.7 | 66.8±23.9 |
|  |  |  |  |  |  |  |  |  |  |  |
| Mother infanT Interaction variables | | | | | | | | | | |
|  | **Baseline**  **(M±SD)** |  | **Week**  **10**  **(M±SD)** |  | **Week**  **36**  **(M±SD)** | **Baseline**  **(M±SD)** |  | **Week**  **10**  **(M±SD)** |  | **Week**  **36**  **(M±SD)** |
| Dyadic synchrony | 6.88±2.74 |  | 7.87±2.99 |  | 9.14±2.81 | 6.71±2.81 |  | 7.92±3.19 |  | 8.64±2.64 |
|  |  |  |  |  |  |  |  |  |  |  |
| maternal unresponsiveness | 3.85±3.55 |  | 2.73±2.86 |  | 2.73±2.81 | 3.42±3.5 |  | 2.07±2.75 |  | 2.0±3.01 |
|  |  |  |  |  |  |  |  |  |  |  |
| infant compulsiveness | 1.74±2.83 |  | 2.22±3.14 |  | 1.33±2.53 | 3.14±2.95 |  | 2.92±3.56 |  | 2.21±2.51 |
|  |  |  |  |  |  |  |  |  |  |  |
| infant passiveness | 2.84±2.78 |  | 1.77±2.54 |  | 1.65±2.41 | 2.28±2.81 |  | 1.92±2.86 |  | 0.71±1.43 |
|  |  |  |  |  |  |  |  |  |  |  |
